# Characterization of the complete mitochondrial genome of the Lesser Spotted Woodpecker (*Dryobates minor*) and its phylogenetic position

**DOI:** 10.1080/23802359.2022.2110530

**Published:** 2022-08-18

**Authors:** Junda Chen, Dehuai Meng, Yuhui Si, Meichen Yu, Liwei Teng, Zhensheng Liu, Xiaoyu Zhou

**Affiliations:** a College of Wildlife and Protected Area, Northeast Forestry University, Harbin, China; b Key Laboratory of Conservation Biology, National Forestry and Grassland Administration, Harbin, China; c The Siberian Tiger Park, Harbin, China

**Keywords:** Complete mitochondrial genome, *Dryobates minor*, woodpecker

## Abstract

In this study, we sequenced and assembled the complete mitochondrial genome of *Dryobates minor* by next-generation sequencing. The mitochondrial genome of *Dryobates minor* is 16,847 bp in length and consists of 13 protein-coding genes (PCGS), two ribosomal RNA (rRNA) genes, 22 transfer RNA (tRNA) genes and 1 control region (CR). The CG content of the mitochondrial genome is 47.46%. Only one overlap among the 13 protein-coding genes was found: ND4L/ND4. Phylogenetic analysis based on a combined mitochondrial gene dataset indicated that the mitochondrial genome of *Dryobates minor* exhibited a close relationship with that of *Picoides pubescens*.

The Lesser Spotted Woodpecker (*Dryobates minor*) (Linnaeus, 1758) plays an important role in both forest structure and relative position (Miranda and Pasinelli 2001) and is widely distributed in Asia and Europe and also occurs in North Africa (Fagan and Holmes 2006). It is the smallest European woodpecker, and it may at once be identified by the broad barring on the wings and narrower bars across the lower back. The *D. minor* and other woodpecker species distributions can often reflect the interplay between habitat availability and climate (Virkkala et al. 2005; Heikkinen et al. 2007; Luoto et al. 2007). Due to the loss of forest biodiversity and disruptions of ecosystem function, the population of *D. minor* has decreased in recent years(Chapin Iii et al. 2000; Kok et al. 2018; Orlikowska et al. 2020). In addition to a trend toward warming springs, a decline in the number of low productivity has become a widespread problem for the *D. minor* (Smith and Smith 2020).

The complete mitochondrial genome of the *D. minor* was sequenced using muscle tissue collected from an individual collected at Heilongjiang, China (127°34′E, 45°17′N). The specimen was deposited at College of Wildlife and Protected Area, Northeast Forestry University (Zhensheng Liu, zhenshengliu@163.com) under the voucher number XBZMN211120. Based on the high-throughput Illumina Hiseq X platform, total genomic DNA was sequenced. The raw data were assembled using MitoZ (Meng et al. 2019) building contig and scaffold sequences, and results were corrected for final mitochondrial sequences using pilon v1.18 (Walker et al. 2014). Subsequently, the mitogenome was annotated with the MITOS WebServer (Bernt et al. 2013). We used whole genome shotgun (WGS) sequecing with the Illumina HiSeq sequencing platform to construct a library of *Dryobates minor* DNA fragments. Following Sangster and Luksenburg (2021), we verified the identity of our mitogenome sequence of *D. minor* with reference sequences of three commonly used markers in avian systematics: NADH dehydrogenase subunit 2 (ND2, 1041 bp; 550 woodpeckers, incl. one *D. minor*), part of cytochrome c oxidase subunit I (COI, 696 bp; 470 woodpeckers, incl. eight *D. minor*), and cytochrome b (Cyt b, 1141 bp; 115 woodpeckers, incl. two *D. minor*). In each of these analyses, our sequence of *D. minor* clustered with the reference sequences of *D. minor*, indicating that our sample was correctly identified. The sequence was submitted to GenBank with the accession number OL597538.

The mitochondrial genome consists of 13 protein-coding genes, 2 ribosomal RNA genes (*rns* and *rnl*), 22 tRNA genes, and 1 control region (CR). The mitochondrial genome of Dryobates minor is 16,847 bp. The base composition of the *Dryobates minor* mitochondrial genome were as follows: A (28.38%), T (24.06%), G (13.77%), and C(33.79%). The AT content of the mitochondrial genome is 52.44%, which does not has a strong AT nucleotide bias. In 13 protein-coding genes, ten (ND1, ND2, COX2, ATP8, ATP6, COX3, ND4L, ND4, CYTB, ND6) used ATG as start codon, two (COX1, ND5) used with GTG as start codon, one (ND3) used ATA as start codons. Eight (ND2, COX2, ATP8, ATP6, ND3, ND4L, ND4, CYTB) ended with TAA as stop codon, two (ND1, COX1) ended with AGG as stop codon, COX3 ended with CGT as stop codon, ND5 ended with AGA as stop codon and ND6 ended with TAG as stop codon.

We constructed a Maximum Likelihood (ML) phylogeny in MEGA7 (Kumar et al. 2016) with 2,000 bootstrap replicates (Tamura et al. 2011). We constructed a phylogenetic tree for 15 species, with *Strix leptogrammica* (Linnaeus, 1758) as the outgroup. The phylogenetic tree showed that *D. minor* was more closely related to *Picoides pubescens* (Linnaeus, 1766) than to species in the genus Dendrocopos (Figure 1). This agrees with the findings of Fuchs and Pons (2015) and supports the transfer of Lesser Spotted Woodpecker from the genus Dendrocopos to the genus Dryobates (Sangster et al. 2016; Sangster and Luksenburg 2021).The complete mitochondrial genome of *D. minor* present in this study will provide useful genetic data for further phylogenetic and evolutionary analysis for Piciformes.

**Figure 1.**
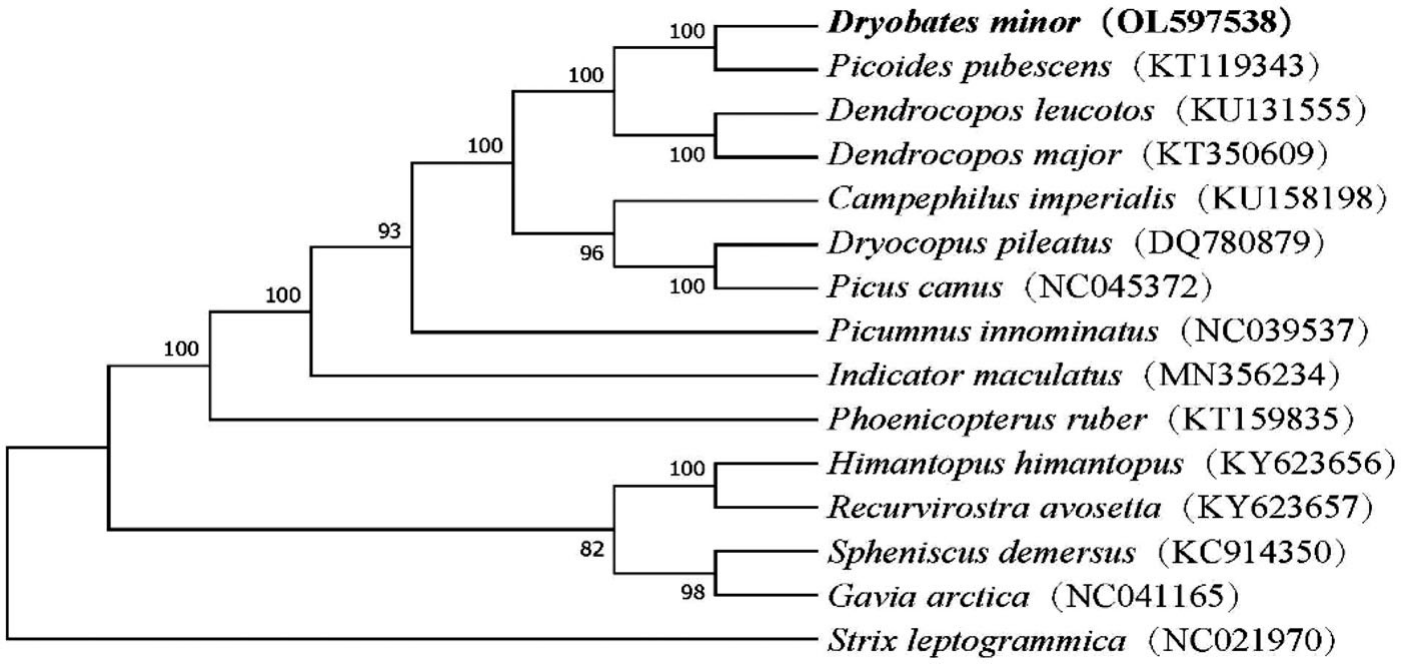
Maximum-likelihood (ML) phylogenetic tree based on the complete mitochondrial genomes of 15 species. *Strix leptogrammica* (Strigiformes) was used as an out-group. Numbers on branches represent bootstrap supports (2000 replicates). GenBank accession numbers for each species are shown in parentheses. The phylogenetic position of *Dryobates minor* was marked with bold.

## Ethical approval

The muscle tissue of Lesser Woodpecker was extracted from the individual which died naturally in the wild not more than 3 days old. The first day of searching in Maorshan yielded no results, while the second yislded a bady. In the scenario, ethical clearance is not necessary.

## Author contributions statement

We thank Professor Zhensheng Liu, Professor Liwei Teng, Dr. Junda Chen, Dr. Dehuai Meng and Xiaoyu Zhou who has been involved in the conception and design of the study and Dr. Yuhui Si, Dr. Meichen Yu for publishing the final approval of the version and Professor Zhensheng Liu and Professor Liwei Teng for collecting the samples and revising the paper critically for intellectual content. All authors agree to be accountable for all aspects of the work.

## Data Availability

The genome sequence data that support the findings of this study are openly available in GenBank of the NCBI at (https://www.ncbi.nlm.nih.gov/) under accession no. OL597538.1. The associated BioProject, SRA, and Bio-Sample numbers are PRJNA781112, SRR16962851 and SAMN23235464, respectively.
